# Pepsin-driven corrosion of orthodontic titanium alloys in candidiasis-simulated saliva: electrochemical and statistical insights

**DOI:** 10.1038/s41598-026-36707-8

**Published:** 2026-02-10

**Authors:** Renad S. El-Kamel, Amany M. Fekry

**Affiliations:** Faculty of Science, Department of Chemistry-Giza, Cairo University, Giza, Egypt

**Keywords:** Saliva, Pepsin, Orthodontic Ti alloy, *Candida albicans*, Response surface methodology, Materials science, Microbiology

## Abstract

Ti-6Al-4 V titanium alloy is widely utilized in orthodontic applications due to its favorable biocompatibility and mechanical properties. However, its long-term performance can be adversely affected by the dynamic and hostile oral environment, particularly under pathological conditions such as gastroesophageal reflux disease (GERD). Herein, In vitro corrosion behavior of Ti-6Al-4 V over a 240-hour immersion period at 37 °C in artificial saliva simulating GERD, with pepsin and *Candida albicans*, both individually and combined. Electrochemical impedance spectroscopy (EIS) and potentiodynamic polarization revealed that pepsin significantly improved corrosion resistance, achieving a maximum inhibition efficiency (IE) of 87.4%, while *C. albicans* showed a time-dependent decline in protection, with lower IE 71.8%. The combined presence of both agents further reduced IE to 55.6%, indicating a complex synergistic effect accelerating corrosion. Surface characterization by scanning electron microscopy (SEM) confirmed biofilm formation and surface degradation. Response Surface Methodology (RSM) modeling identified immersion time and component interactions as key factors influencing corrosion behavior. These findings offer novel insights into the interplay among enzymatic activity and microbial colonization, highlighting clinical implications for implant stability in GERD-affected oral environments.

## Introduction

In the past few decades, titanium alloys have been broadly used in dental prostheses for instance metal plates, orthodontic wires and orthodontic materials, metal-ceramic restorations, crowns, and bridges^1^. Human body susceptibility to hypoallergenic metal implants is a common adverse event, and the ability of FDA-approved titanium devices to tolerate the metal ions created or released during long-term implantation raises ethical considerations^2^. Titanium (Ti) alloys are indispensable in key applications, such as medical devices, owing to their outstanding specific strength and biocompatibility. The notable corrosion resistance of titanium is attributed not only to its inherent material properties and the formation of a stable, self-healing passive oxide layer, but also to its interactions with the surrounding environment^3^.

To ensure biocompatibility, it is essential to understand the corrosion mechanisms of titanium and its alloys within the oral cavity and to develop strategies that minimize the release of potentially harmful metal ions into the human body^4^.

The oral cavity represents a sensitive environment where a delicate equilibrium is maintained between the teeth and surrounding tissues. Saliva composition and pH vary among individuals, as saliva is a complex fluid containing inorganic salts, acids, enzymes, bacteria, and gastric secretions^5^. The oral microbiota comprises diverse microorganisms, including bacteria, fungi, protozoa, mycoplasma, and viruses^6^, which exist in a mutually regulated and interdependent relationship to sustain the homeostasis of the oral microbiome^7^.

*Candida* species are opportunistic fungi commonly present in the environment as well as on human skin and mucosal surfaces. While typically harmless, they can proliferate rapidly and form mycelial structures under specific conditions, leading to pathogenic infections^8– 9^.

*Candida albicans* is responsible for approximately 25–75% of oral candidiasis cases and affects up to 90% of the elderly population. Its pathogenicity is largely attributed to its remarkable ability to adhere to surfaces. This adhesion is further facilitated by the hydrophobic properties and electrostatic interactions of its cell surface^10^.

Gastroesophageal reflux disease (GERD) is a common chronic condition characterized by the reflux of gastric contents, including hydrochloric acid and bile, into the esophagus. This backflow leads to mucosal injury and symptoms such as heartburn, regurgitation, and dysphagia^11– 12^. Recently, salivary pepsin has gained attention as a potential biomarker for the diagnosis of GERD and other acid-related disorders^13^.

Pepsin is one of the primary proteolytic enzymes responsible for protein digestion within the digestive system^14– 15^. Gastric juice, characterized by a highly acidic pH ranging from 1 to 3, contains hydrochloric acid (HCl), chloride ions (Cl⁻), and pepsin^16^. In contrast, intestinal juice has a near-neutral pH between approximately 5.0 and 7.7, achieved through neutralization of gastric acid by pancreatic secretions and buffering agents to protect the intestinal mucosa from acidic damage^17^.

Saliva, the primary aqueous component of the oral cavity, becomes more acidic in the presence of GERD, with pH values dropping to around 4.9 compared to the near-neutral pH of 6.5 observed in healthy individuals^18^. The inclusion of *Candida albicans* in this study is justified by its prominent role as a pathogen in oropharyngeal candidiasis, particularly among patients with orthodontic devices. Investigating the influence of pepsin on corrosion and biofilm development on titanium alloys offers critical insights into the complex interplay between microbial infections, enzymatic activity, and the oral environment, and their combined impact on the durability of orthodontic materials.

**Fig. 1:**
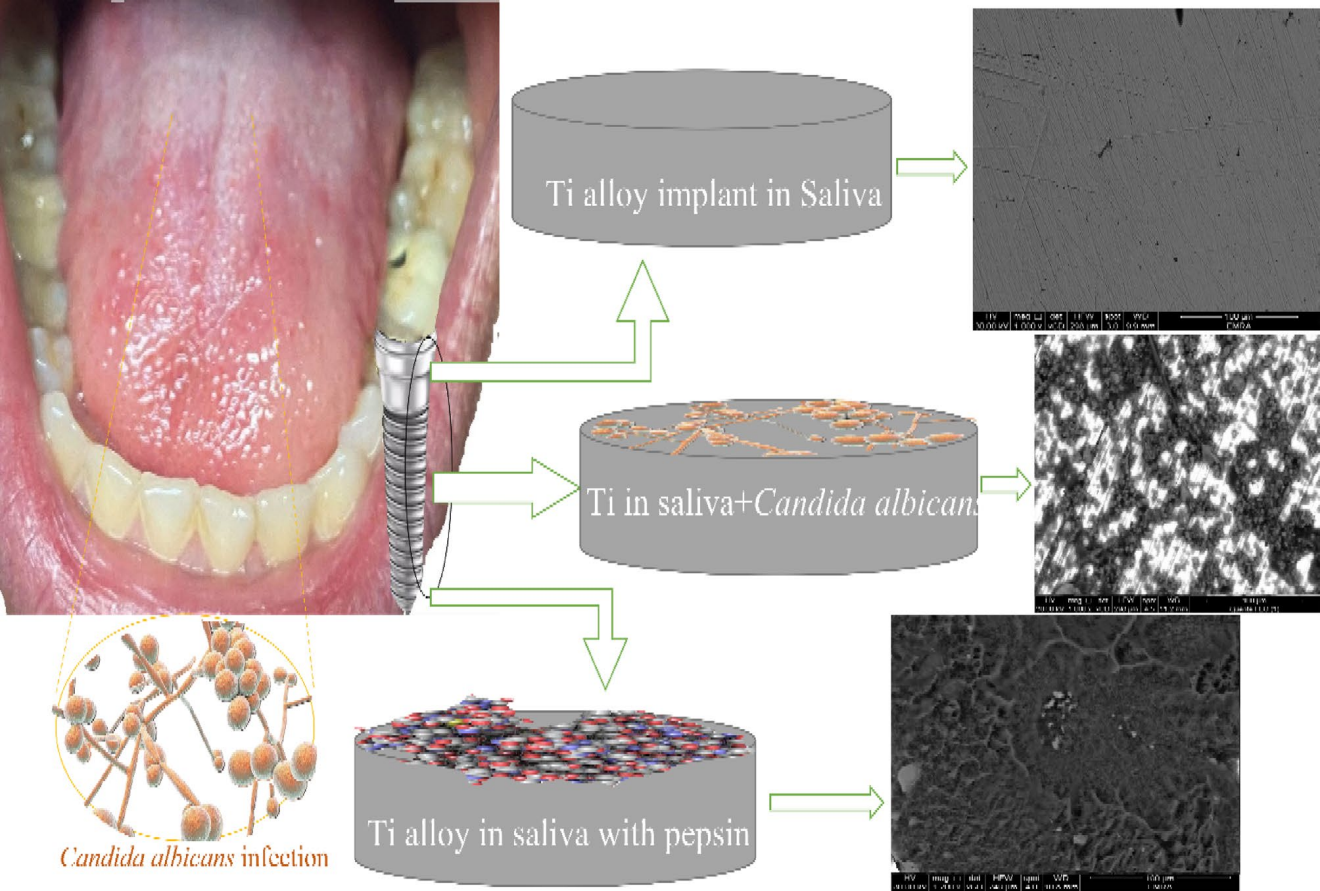
A schematic diagram of the aim of this work. (Note: This figure was created by the authors. The mouth image is an original photograph taken by the authors specifically for this research, and no copyrighted third-party material was used).

This study (Fig. 1) evaluates the electrochemical behavior of the Ti–6Al–4 V alloy in artificial saliva, with a particular focus on the impact of *Candida albicans* as a biological contaminant. The influence of pepsin—a digestive enzyme frequently present in the oral environment under gastroesophageal reflux disease (GERD) conditions—was also assessed at 37 °C across different immersion periods. Corrosion inhibition efficiency was quantified as a function of immersion time. Additionally, Response Surface Methodology (RSM) was utilized to model and optimize the corrosion response, given its demonstrated effectiveness in simplifying and predicting complex corrosion phenomena^19^.

## Experimental procedure

### Solution preparation and electrode composition

Pepsin was sourced from Loba Chemie and prepared in a solution adjusted to pH 2.5. To clarify the final pH of the GERD-simulated solution, pepsin was first dissolved in distilled water and adjusted to pH 2.5 to ensure enzymatic activation. This pepsin stock solution was then mixed with artificial saliva (initial pH 6.5) in the predetermined volume ratio. Upon mixing, the solution equilibrated to a physiologically relevant GERD-simulated pH of approximately 4.9 as a result of the interaction between the acidity of pepsin and the buffering capacity of artificial saliva. The final pH (4.9 ± 0.1) was verified using a calibrated pH meter prior to initiating all corrosion measurements. The pH was carefully controlled using 0.1 M NaOH or 0.1 M HCl, measured with a HANNA 213 pH meter. The Ti-6Al-4 V alloy utilized in this study, supplied by Johnson and Matthey (England), has its elemental composition (wt%) presented in Table 1. Cylindrical specimens of the alloy, each with an exposed surface area of 0.196 cm², were prepared according to protocols outlined in our previous research.

**Table 1:** Alloy composition^20^.

Composition	Al	V	Fe	C	O	*N*
Wt. %	5.7	3.85	0.18	0.038	0.106	0.035

Artificial saliva (AS) was formulated using high-purity (analytical grade) chemicals with the following concentrations (g/L)^21^: 0.72 KCl, 0.22 CaCl₂·2 H₂O, 0.60 NaCl, 0.68 KH₂PO₄, 0.866 Na₂HPO₄·12 H₂O, 1.50 KHCO₃, 0.06 KSCN, and 0.03 citric acid. The pH of the solution was adjusted to 6.5 using triply distilled water.

### Fungal culture media

*Candida albicans*, obtained from the National Collection of Industrial Microorganisms, was maintained on an agar-based culture medium containing (g/L): glucose (10), peptone (10), yeast extract (5), and agar (10–20), and stored at 4 °C. A microbial smear was subsequently introduced into the artificial saliva solution for testing. The solution without microbial inoculation served as the control. The *Candida albicans* inoculum was prepared and standardized using the same methodology reported in our previous work^16^, ensuring consistency and reproducibility.

The sterilized orthodontic Ti–6Al–4 V alloy, serving as the working electrode, was immersed in artificial saliva at 37 °C under anaerobic conditions for varying durations (1 h, 24 h, 48 h, 96 h, 168 h, and 240 h). Following each immersion period, electrochemical impedance spectroscopy (EIS) and potentiodynamic polarization measurements were performed from − 1.2 to 0 V at a scan rate of 1 mV·s⁻¹. Electrochemical analyses were conducted using an SP-150 potentiostat (EC-Lab^®^ software, BioLogic), employing its integrated software for data acquisition and curve fitting. A conventional three-electrode setup was used under conditions consistent with those described by El-Kamel et al.^15,22^. All electrochemical experiments were repeated in triplicate to ensure reproducibility and statistical robustness, with results reported as mean values and standard deviations where applicable.

To optimize and model the inhibition efficiency, Response Surface Methodology (RSM) was applied using a Central Composite Design (CCD) for experimental planning. Data analysis was performed with Minitab 19 software, and Analysis of Variance (ANOVA) was used to evaluate the statistical significance of the independent variables (immersion time and component presence) and their interaction effects. A second-order polynomial (quadratic) model was developed to predict the inhibition efficiency, with model adequacy assessed via the coefficient of determination (R²) and adjusted R-squared (R²_adj) values.

### Surface characterization

Surface characterization of the Ti–6Al–4 V alloy was performed using a Quanta 250 FEG Scanning Electron Microscope (Field Emission Gun; FEI Company, Netherlands). The instrument operated at an accelerating voltage of 30 kV, offering magnifications ranging from 14X to 1,000,000X and a resolution of 1 nm.

## Results and discussion

### Characterization of Ti alloy electrode

**Fig. 2:**
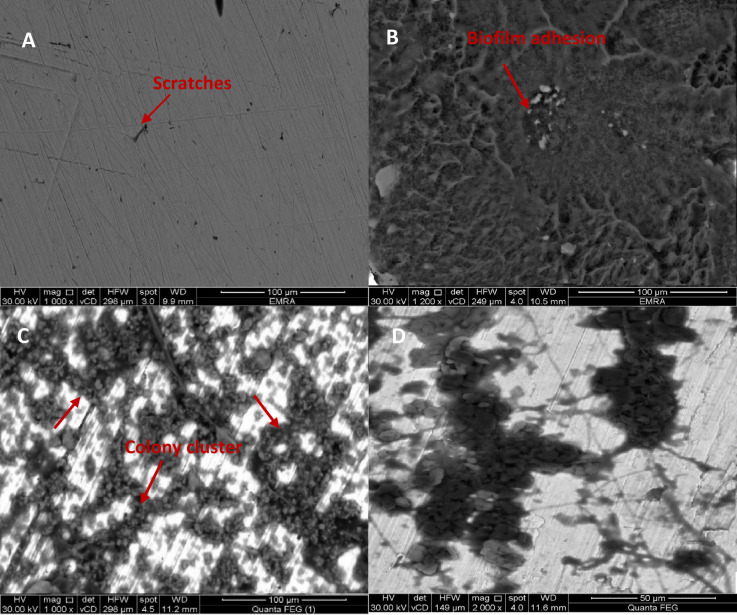
SEM images of (**A**) bare Ti-6Al-4 V alloy without and with (**B**) pepsin and (**C**)* C.albicans* and (**D**) at higher magnification of *C.albicans*.

Figure 2. of SEM characterization reveals the surface morphology of Ti-6Al-4 V alloy as bare in Fig.2A with the absence of pepsin in Fig. 2B in the presence of pepsin and microbial group after immersion time. A compact biofilm formed due to the presence of pepsin as a protein in artificial saliva solution (Fig. 2B). Non-uniform *C. albicans* colony adsorption is observed at the alloy surface (Fig. 2C and D), with Fig. 2D showing a higher magnification (2000x) of *C. albicans* presence.

Key morphological features such as scratches, biofilm clusters, and corrosion-affected zones were visually annotated in Fig. 2 to facilitate interpretation of surface changes under different exposure conditions.

### Electrochemical impedance spectroscopy (EIS) measurements

**Fig. 3:**
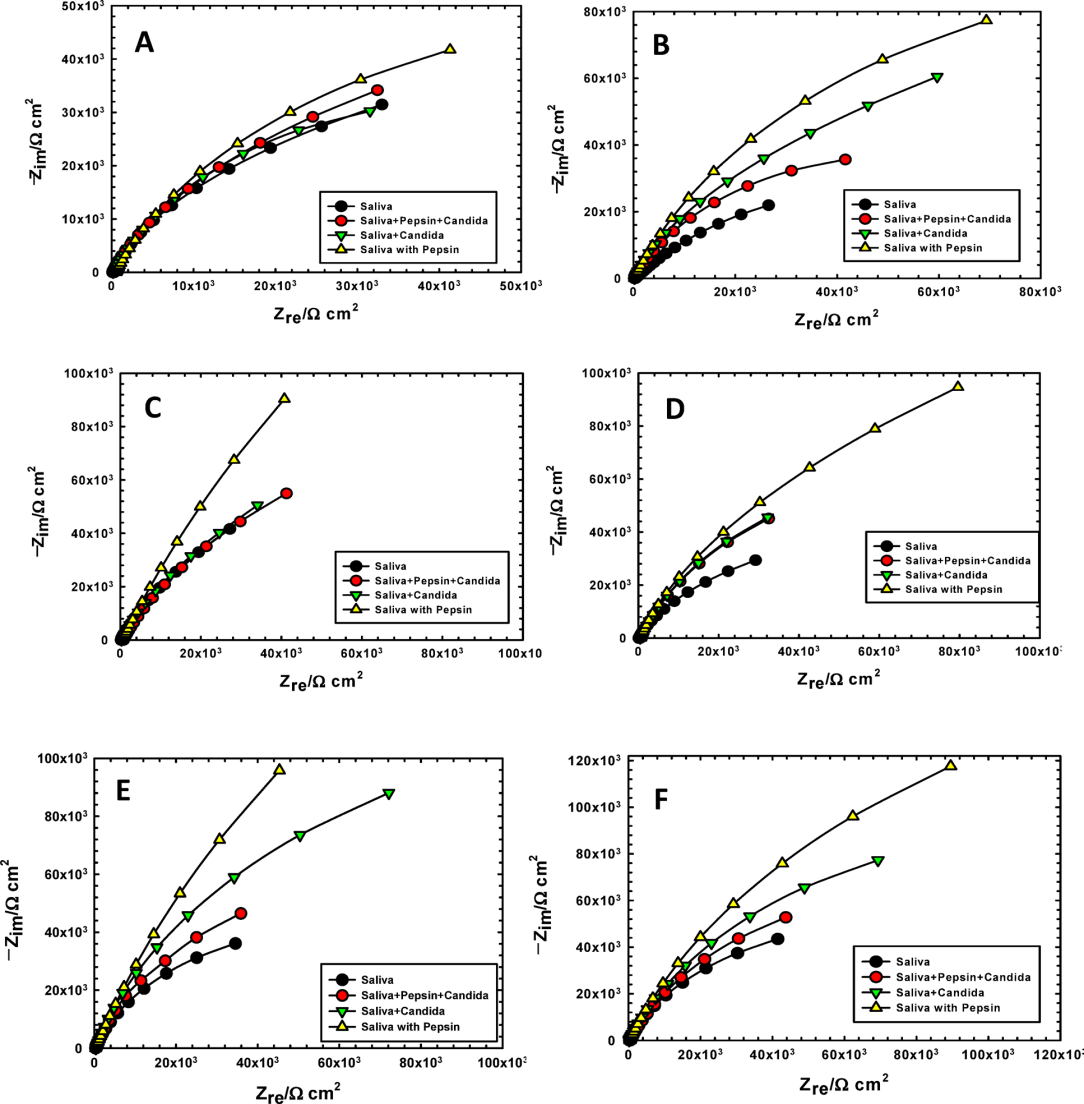
Nyquist plots of (a) Ti-6Al-4Valloy in saliva without and with pepsin and *Candida albicans* after immersion for (**A**) 1 h, (**B**) 24 h, (**C**) 48 h, (**D**) 96 h, (**E**) 168 h and (**F**) 240 h. Starting and ending frequencies (0.1 Hz to 100 kHz).

**Fig. 4:**
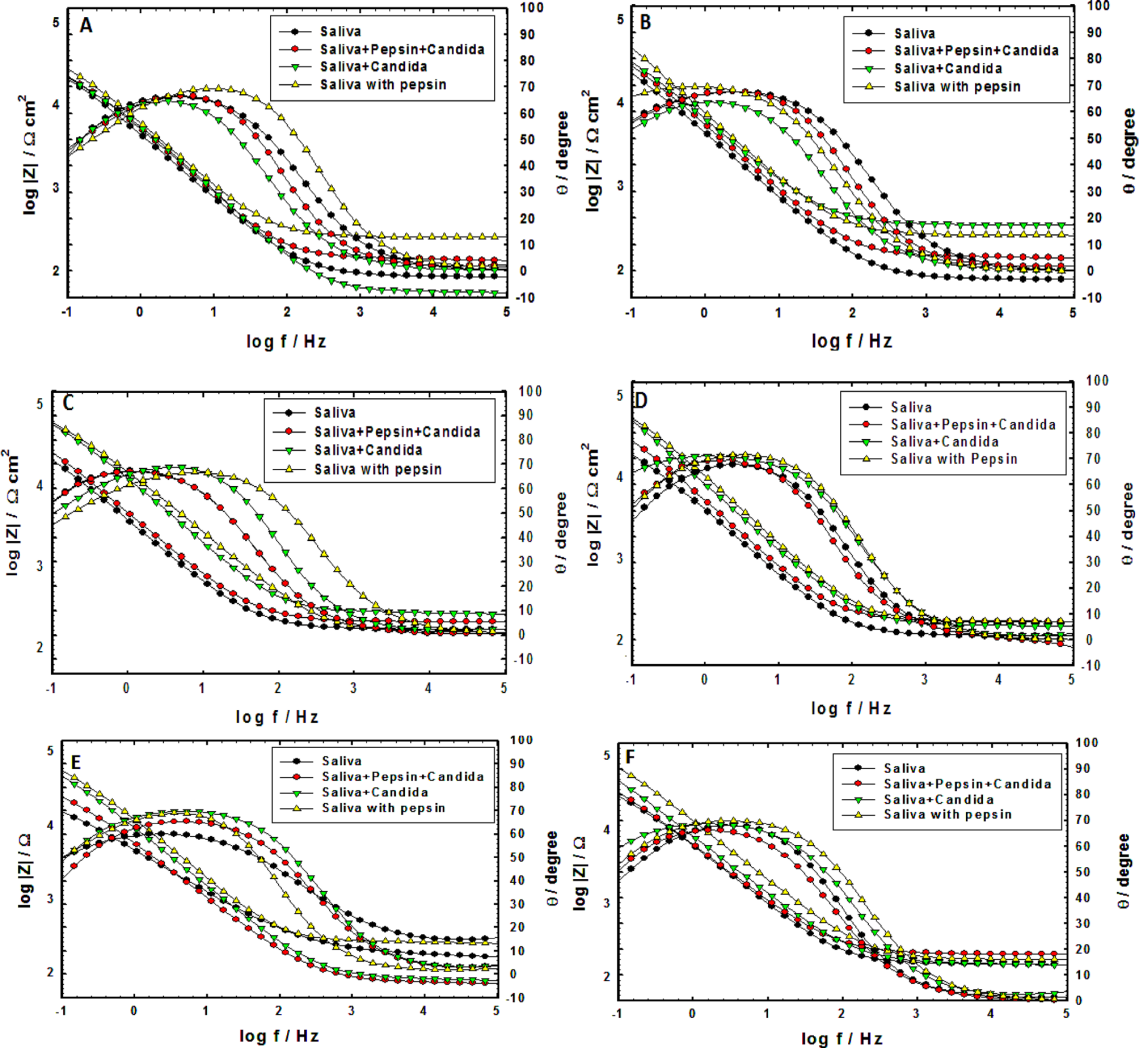
Bode plots of (a) Ti-6Al-4 V alloy in saliva without and with pepsin and *Candida albicans* after immersion for (**A**) 1 h, (**B**) 24 h, (**C**) 48 h, (**D**) 96 h, (**E**) 168 h and (**F**) 240 h.

Figures 3 and 4 display the Nyquist and Bode plots derived from electrochemical impedance spectroscopy (EIS) of orthodontic Ti-6Al-4 V alloy samples immersed in artificial saliva, both with and without the presence of pepsin and *Candida albicans*, individually and combined. The impedance spectra consistently demonstrate pronounced capacitive behavior, with phase angles approaching a maximum near 80°^23^.

The Nyquist plots (Fig. 3) consistently exhibit incomplete semicircles, where a larger semicircle diameter corresponds to enhanced corrosion resistance. The data reveal that samples exposed to pepsin demonstrated superior corrosion resistance and inhibition compared to those with *Candida albicans*, which is characterized by its vigorous metabolic activity. Complementary Bode plots (Fig. 4) indicate that pepsin, as a protein, plays a significant chelating and barrier role, influencing the corrosion kinetics of the titanium alloy. This corrosion inhibition is attributed to the physical adsorption of pepsin molecules onto the alloy surface^24]– [25^. Furthermore, the protective blocking effect of pepsin intensifies with prolonged immersion time, effectively reducing the corrosion rate of the Ti-6Al-4 V alloy^26^.

Accumulation of biofilm formed by *Candida albicans* metabolites—including polysaccharides, proteins, and organic acids—enhances cellular adhesion and cohesion on both inert and active surfaces, facilitating stable hyphal attachment to the metal substrate.

A minor disruption was observed when *Candida albicans* coexisted with pepsin in the saliva solution, potentially reflecting conditions within the oral cavity where various microbial species can influence titanium-containing materials^27–29^. Orthodontic appliances provide an ideal surface for *C. albicans* adhesion and biofilm development, involving complex interactions. Pepsin actively inhibits the attachment of pathogenic microorganisms, alters local pH and microbial habitat, secretes antimicrobial agents, and modulates the host’s immune response. Additionally, fungal metabolic byproducts in the presence of pepsin may modify the microenvironment, potentially contributing to the corrosion of titanium alloys.

Previous studies^30^ have demonstrated that solutions with pH values below 2.5 exhibit significant antimicrobial activity against fungi, bacteria, and various microorganisms, with this effect diminishing at pH 3.0 and becoming negligible above pH 3.5. Additionally, research indicates no bactericidal effect within the pH range of 4.0 to 7.0^31^. However, in the oral cavity affected by gastroesophageal reflux disease, the buffering action of pepsin elevates the pH to approximately 4.5, considerably higher than the stomach environment. Moreover, bacteria may be protected from acidic conditions by binding to pepsin, suggesting that the stomach’s acidic milieu does not effectively eradicate these microorganisms under such circumstances.

**Fig. 5:**
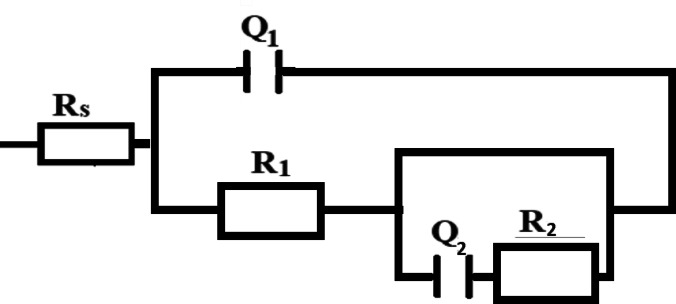
Equivalent circuit model used for fitting.

The experimental EIS data were analyzed by fitting to the equivalent electrical circuit depicted in Fig. 5. This circuit models the oxide film on the Ti-6Al-4 V alloy as a bilayer structure comprising an outer and an inner layer. In the model, Rs denotes the solution resistance, R1 represents the resistance of the outer oxide layer, and R2 corresponds to the charge transfer resistance at the inner layer. The elements Q1 and Q2 are constant phase elements (CPEs) associated with the double-layer capacitance and the passivation film, respectively. Notably, the charge transfer resistance R2 serves as a critical parameter directly related to the corrosion resistance of the alloy. The double-layer equivalent circuit model shown in Fig. 5 (R₁/Q₁ for the outer layer and R₂/Q₂ for the inner layer) was selected because titanium alloys typically form a duplex passive film consisting of an outer porous/hydrated oxide layer and an inner compact TiO_2_ barrier layer^32-33^.

The constant phase element (CPE) was employed instead of an ideal capacitor (C) to account for the surface heterogeneity and roughness of the alloy. The fitting parameters derived from this model, as detailed in Table 2, accurately represent the electrochemical behavior observed during the measurements^34^:

1 $$Z_{{CPE}} = \frac{1}{{Q\left( {j\omega } \right)^{\alpha } }}$$

where Q is the magnitude of the CPE, j is the imaginary unit, ω is the angular frequency, and α is the CPE exponent. The fitting data is given in Table 2.

The alloys immersed in the simulated oral environment exhibited a passive state throughout the study. The increase in impedance values indicates an enhancement in the electrochemical resistance of the passive film under the combined action of pepsin and *Candida albicans*, suggesting improved protective behavior. The solution resistance (Rs) remained relatively low, attributable to the high ionic content of the saline medium. The increased resistance values indicate that biofilm formation contributed to the overall enhancement of the impedance response.

The inhibition efficiency is estimated from^35– 36^:

2 $$\:IE\%=\frac{{R}_{T}-{R}_{T}^{o}}{{R}_{T}}\times\:100$$

Where $\:{R}_{T}^{o}$ and$\:\:{R}_{T}\:$represnt the total resistances of unexposed Ti and exposed Ti alloys to pepsin, *Candida albicans;* and both, respectively.

The inhibition efficiency of the titanium alloy progressively increased during immersion in artificial saliva containing pepsin, reaching a maximum of 87.4% after 240 h. In contrast, the combined presence of *Candida albicans* and pepsin in saliva resulted in a reduced inhibition efficiency of 55.6% at the same duration. Samples exposed to *C. albicans* alone in saliva maintained effective inhibition up to 96 h, after which a decline was observed, suggesting that while *C. albicans* initially contributes to surface protection, prolonged exposure may promote degradation. These findings imply a complex interplay between pepsin and *C. albicans*, where the protective influence of pepsin is attenuated by microbial activity over time. Moreover, *C. albicans* may evade low pH stress by binding to pepsin, thereby modulating its role in the corrosion behavior of the titanium alloy.

In the absence of protective agents, such as in saliva alone, the titanium alloy is directly exposed to the corrosive electrolyte, resulting in the highest corrosion rate due to the lack of any protective barrier film. Conversely, when pepsin or *Candida albicans* is present individually, the titanium surface experiences enhanced protection compared to their combined presence or saliva alone.

Pepsin, when present alone in artificial saliva, likely adsorbs onto the alloy surface forming a proteinaceous film that acts as a barrier, limiting the diffusion of corrosive ions toward the metal interface. Similarly, *C. albicans* alone forms a microbial biofilm on the titanium surface, serving as a physical shield that impedes the transport of aggressive species and restricts anodic dissolution. The reduction in anodic corrosion current observed in these cases indicates that both films effectively inhibit the anodic metal oxidation reaction, thereby retarding the corrosion rate.

However, the simultaneous presence of pepsin and *C. albicans* diminishes this protective effect, as evidenced by a more negative corrosion potential and elevated corrosion current density. This decline in inhibition may result from biochemical interactions in which pepsin’s proteolytic activity degrades components of the microbial biofilm matrix or compromises microbial cell integrity, leading to fragmentation or destabilization of the biofilm barrier. Additionally, metabolic byproducts of *C. albicans* in the presence of pepsin, such as organic acids, could locally acidify the environment, further accelerating the corrosion process by intensifying the aggressive conditions at the metal surface.

**Table 2:** The impedance parameters of immersed bare Ti-6Al-4 V alloy in artificial saliva solution with additives at 37 ◦C.

Ti alloy + Solution	Time h	*R*_s_ Ω cm^2^	*R*_1_ Ω cm^2^	α_1_	Q_1_ µF cm^− 2^	R2 MΩ cm^2^	α_2_	Q_2_ µF cm^− 2^	EI%
Artificial Saliva	1 h	26.7	25.0	0.89	13.14	0.8	0.84	5.0	-
	24	18.2	48.7	0.91	11.21	1.6	0.86	2.5	-
	48	18.5	58.0	0.93	11.04	1.8	0.83	2.4	-
	96	19	67.5	0.91	10.83	1.9	0.81	2.0	-
	168	19.5	73.8	0.92	9.12	2.0	0.82	2.1	-
	240	22.1	82.0	0.93	8.36	2.1	0.82	1.9	-
Saliva + *C.albicans *+ Pepsin	1 h	281.6	131.0	0.94	6.82	2.02	0.83	20.4	60.4
	24	299.4	232.2	0.96	6.65	2.32	0.75	18.1	31.1
	48	446.7	397.8	0.97	5.77	2.67	0.83	17.6	32.6
	96	347.6	470.8	0.96	4.71	3.61	0.81	16.5	47.4
	168	175.7	509.7	0.99	2.06	3.94	0.76	14.9	49.2
	240	402.2	513.8	0.97	2.3	4.73	0.79	14.6	55.6
Saliva + *C.albicans *	1 h	114.7	241.5	0.95	20.49	2.57	0.93	19.73	68.8
	24	738.7	342.7	0.94	16.28	4.65	0.99	19.42	65.6
	48	557.3	398.4	0.96	5.27	9.89	0.82	18.12	81.7
	96	308.1	485.0	0.95	5.49	9.56	0.81	17.42	80.1
	168	211.1	599.7	0.84	5.97	8.36	0.77	16.39	76.1
	240	309.3	711.4	0.92	6.39	7.45	0.81	14.81	71.8
Saliva + Pepsin	1 h	527.3	419.2	0.76	16.8	4.27	0.96	7.50	81.3
	24	560.1	698.1	0.81	13.4	8.94	0.83	9.89	82.1
	48	355.4	738.6	0.83	10.3	11.00	0.99	8.56	83.6
	96	365.3	892.2	0.82	7.85	12.99	0.99	4.68	85.4
	168	579.1	987.8	0.78	7.04	14.20	0.97	2.12	85.9
	240	364.6	996.0	0.89	2.80	16.73	0.95	1.98	87.4

### Potentiodynamic polarization measurements

**Fig. 6:**
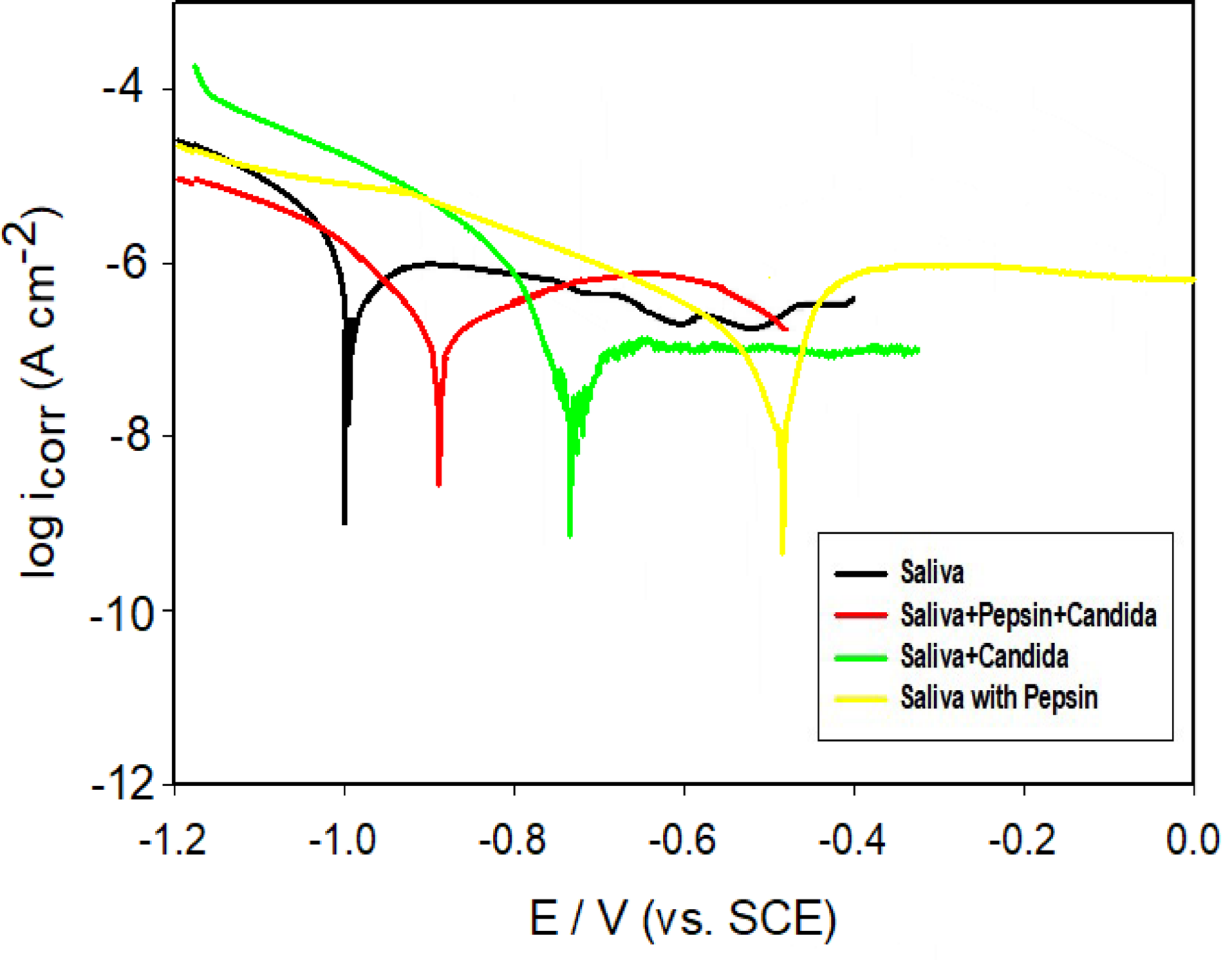
Tafel plots for bare Ti alloy after immersion in artificial saliva solution with pepsin,*C. albicans* and both at 37 °C.

To validate the EIS findings, Tafel polarization curves were recorded for bare Ti-6Al-4 V alloy immersed in artificial saliva containing pepsin, *Candida albicans*, and their combination after 10 days at 37 °C. Measurements were conducted within a potential range of − 1.2 to 0 V at a scan rate of 1 mV·s⁻¹. The resulting Tafel plots are presented in Fig. 6, while the derived polarization parameters—including corrosion potential (E_corr_) and corrosion current density (I_corr_), obtained by extrapolating the linear regions of the curves—are summarized in Table 3^37^. A trend of increasingly positive corrosion potentials was observed in the following order: pepsin + saliva > *C. albicans* + saliva > pepsin + *C. albicans* + saliva > saliva alone. Notably, the anodic corrosion currents were lower in the presence of either *C. albicans* or pepsin alone compared to the bare alloy and the combined presence of both agents, indicating superior corrosion inhibition under single-component exposure. This enhanced protection is likely attributable to the formation of a microbial or proteinaceous biofilm on the titanium surface, effectively reducing corrosion when either *C. albicans* or pepsin is present individually. These observations align well with the results obtained via EIS analysis. Furthermore, the most positive corrosion potential and minimal cathodic hydrogen evolution were recorded for the pepsin-only condition, confirming its significant protective effect as also evidenced by the EIS data.

In this study, artificial saliva containing *Candida albicans* exhibited the lowest corrosion current density of 79 ± 16 nA cm⁻² after 10 days of immersion, indicating a notably strong biological corrosion inhibition.

**Table 3:** Corrosion parameters (mean Value ± SD) of immersed bare Ti alloy in artificial saliva solution with and without addition for 240 h at 37 ◦C.

Medium	E_corr (_mV vs. SCE)	i_corr_ / nA cm^− 2^
Artificial Saliva	-1004 ± 35	239 ± 11
Saliva + Pepsin + *Candida*	-880 ± 10	112 ± 23
Saliva + *Candida albicans*	-715 ± 5	79 ± 16
Saliva with Pepsin	-490 ± 9	89 ± 11

The corrosion characteristics of Ti-6Al-4 V alloy immersed in artificial saliva are significantly affected by the presence of pepsin and *Candida albicans*. These entities influence corrosion through intertwined biochemical and electrochemical mechanisms. Pepsin molecules adhere to the titanium alloy surface, creating a protective proteinaceous layer. This film serves as a physical barrier that impedes the ingress of corrosive ions such as chloride (Cl⁻) and protons (H⁺), thereby limiting their interaction with the metal substrate. This adsorption process can be generalized as follows:

$$Ti{\text{ }}alloy_{{surface}} + {\text{ }}Pep\sin _{{solution}} \to Ti - pep\sin _{{adsorbed}}$$

The formation of this adsorbed layer obstructs anodic sites on the titanium surface, reducing the rate of metal dissolution and subsequently decreasing the corrosion current density.

Separately, *C. albicans* cells attach to the alloy surface and secrete extracellular polymeric substances (EPS), which mainly consist of polysaccharides, proteins, and organic acids. This secretion leads to the establishment of a biofilm:

$$Ti{\text{ }}alloy_{{surface}} + C.albicans{\text{ }}cells \to {\text{ }}Ti - Biofilm$$

The biofilm acts as a physical shield and alters the immediate microenvironment at the metal interface, potentially lowering corrosion by restricting the diffusion of aggressive ions.

The coexistence of pepsin and *C. albicans* results in an antagonistic synergy by turning the protective agents against one another. The synergistic acceleration of corrosion by pepsin and Candida albicans is driven by interfacial enzymatic degradation. Pepsin’s proteolytic activity compromises the protective fungal biofilm barrier, simultaneously releasing smaller peptides and acidic degradation products that act as corrosion promoters. This combined action creates a highly electrochemically active interface, which intensifies degradation and results in significantly higher overall corrosion compared to independent exposure conditions. The proteolytic function of pepsin at the simulated GERD pH actively and enzymatically degrades the structural polymers (proteins and polysaccharides) within the biofilm matrix:

$$Biofilm~matrix_{{\left( {EPS} \right)}} ~ + ~pep\sin _{{\left( {enzyme} \right)}} \mathop \to \limits^{{proteolysis}} peptides{\text{ }} + {\text{ }}a\min o{\text{ }}acids$$

This biochemical attack severely destroys the physical barrier function of the biofilm, immediately exposing the underlying titanium passive film to corrosive agents and driving the accelerated corrosion kinetics observed in the combined system.

### Multivariate optimization

#### Analysis and optimization based on RSM methodology

The influence of pepsin and *Candida albicans* on inhibition efficiency was evaluated using Response Surface Methodology (RSM), a statistical and mathematical approach designed to model and analyze systems in which multiple variables influence a given response. Prior to applying RSM, a structured experimental design is required. In this study, a Central Composite Design (CCD) was employed, offering an efficient means of assessing potential interactions between variables and identifying optimal conditions, while minimizing the number of experimental trials and resource consumption^38^.

The experimental data obtained were analyzed using Minitab 19 software to perform an analysis of variance (ANOVA). Various predictive models, expressed in terms of coded factors, were developed to estimate the response values at specific levels of each variable. A three-level Central Composite Design (CCD) with two independent factors coded as − 1, 0, and + 1 was employed in this study. This design efficiently reduced the total number of experimental runs to 13, encompassing different point types for accurate model generation and response prediction.

**Table:** 

Cube points:	4
Center points in cube:	5
Axial points:	4
Center points in axial:	0

The factor levels along with their corresponding actual values are summarized in Table 4, while the design of experiments (DOE), including both actual and coded values, is detailed in Table 5.

**Table 4:** Experimental range of independent variables.

Variable	Symbol	Low level	Average level	High level
Time, h	A	1 (-1)	120 (0)	240 (+ 1)
Components existence	B	*Candida albicans* (C)(-1)	*Candida albicans* + Pepsin (C + P) (0)	Pepsin (P)(+ 1)

**Table 5:** RSM results of the Inhibition efficiency of components and time on Ti alloy.

Run order	Coded variables		Uncoded factors values		Response % IE%
	A	B	Time, h	Addition components	
1	0	0	120	C + P	58.0
2	0	-1	120	C	70.3
3	0	0	120	C + P	58.0
4	-1	1	1	P	81.3
5	0	0	120	C + P	57.9
6	0	1	120	C	69.9
7	1	-1	240	P	87.4
8	1	0	240	C + P	55.6
9	0	0	120	C + P	58.2
10	-1	0	1	C + P	60.4
11	0	0	120	C + P	57.8
12	1	1	240	P	87.4
13	-1	-1	1	C	68.8

In the present study, Response Surface Methodology (RSM) was employed to investigate the interactive effects between the experimental variables, namely immersion time and component presence.

Analysis of Variance (ANOVA) was applied to identify the design parameters that significantly affect the inhibition efficiency. ANOVA also served to determine which model terms had a statistically meaningful influence on the experimental outcomes. Based on the Central Composite Design (CCD), the ANOVA results for the response surface model indicated that a quadratic model was well-suited for analyzing the experimental data, as summarized in Table 6.

The F-value (Fisher ratio) and the p-value are key indicators of the overall statistical significance of the model. A high F-value relative to a low p-value suggests that the model reliably explains the variability in the response and that the null hypothesis can be rejected. In this study, the model exhibited an F-value of 10.19 and a p-value of 0.004, which is below the significance threshold of 0.05. This confirms that the model is statistically significant and capable of predicting the optimal conditions for achieving maximum inhibition efficiency. Moreover, the quadratic term for factor B² displayed a p-value below 0.05 and an F-value of 29.83, indicating that this parameter has a substantial effect on the inhibition efficiency.

**Table 6:** Analysis of variance (ANOVA) for response surface model.

Source	DF	Sum of squares	Mean square	F-Value	*P*-Value
Model	5	1441.73	288.345	10.19	0.004
A-time/h	1	34.32	34.324	1.210	0.307
B-components	1	63.34	63.339	2.240	0.178
AA	1	79.56	79.560	2.810	0.137
BB	1	844.00	844.001	29.83	0.001
AB	1	38.95	38.946	1.380	0.279
Error	7	198.03	28.291		
Lack-of-Fit	3	197.95	65.982	2999.19	0.000
Pure Error	4	0.09	0.022		
Total	12	1639.76			
S	5.31890				
R-sq	87.92%				
R-sq(adj)	79.30%				

The adequacy of the model was assessed using the coefficient of determination (R²) alongside the adjusted R² value, as recommended in previous studies^39^. The obtained R² values of 79.30% and 87.92% for the inhibition efficiency response indicate a strong correlation and a satisfactory agreement between the experimental results and the values predicted by the model. These results confirm the model’s reliability and its suitability for accurately representing the system’s behavior.

The final regression equation, expressed in terms of coded factors, for predicting the inhibition efficiency is given as follows:

3 $$IE\% = 58.58{\text{ }} - ~0.0628~A{\text{ }} + ~5.16~B{\text{ }} + ~0.000376~A*A{\text{ }} + ~17.48~B*B{\text{ }} - ~0.0261~A*B$$

The final regression equation, expressed in terms of coded factors, for predicting the inhibition efficiency is presented below, where A represents the immersion time (hours) and B denotes the presence of components (pepsin and/or *Candida albicans*).

#### Surface response plots for Inhibition efficiency

**Fig. 7:**
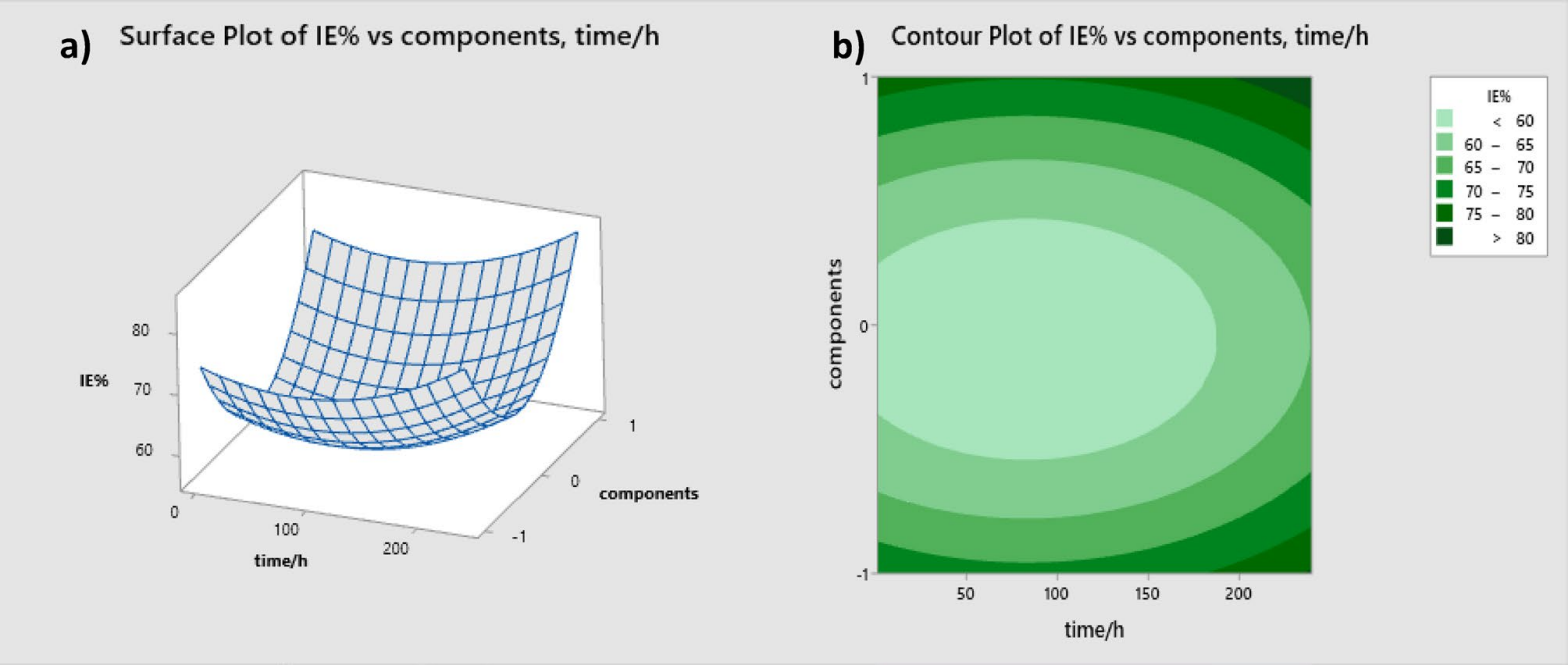
(**a**) 3D response surface plot and (**b**) Contour plot of the combined effect of time and existence components variable on inhibition efficiency.

 The influence of the two factors—component presence and immersion time—on the response variable (IE) is illustrated through the three-dimensional surface plots shown in Figure 7a^40^.

When temperature is preserved persistent, IE increases with accumulative time and in pepsin addition components more than *candida albicans* and IE% decreasing with existence pepsin with candida at the same time. This results also confirmed with Contour plots of the combined effect of time and existence components variable on inhibition efficiency (Fig. 7b).

**Fig. 8:**
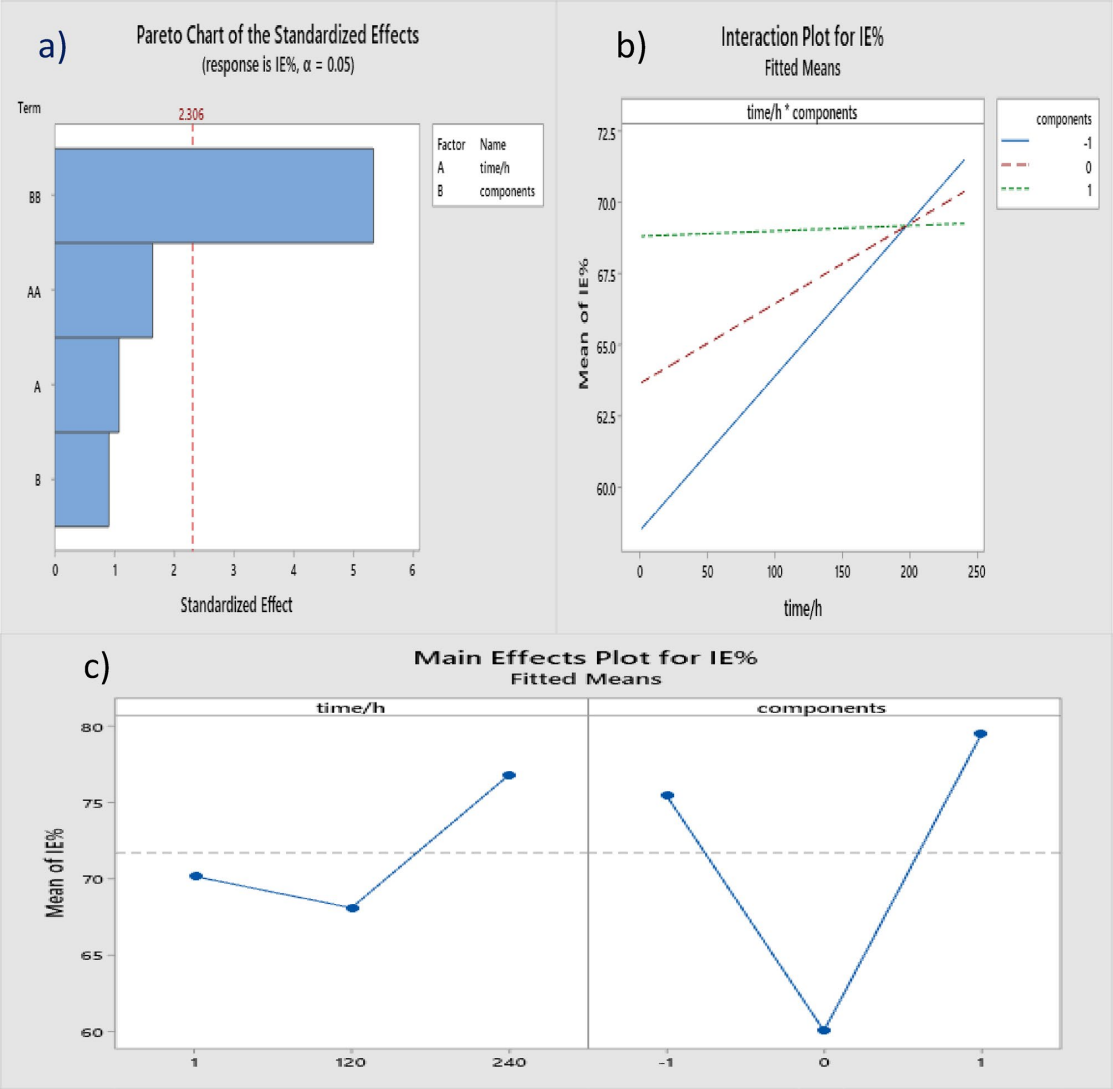
Factorial Plots, (**a**) pareto standardization effect, (**b**) interaction plot and (**c**) main effect of IE% for Ti alloy in pepsin, *C.albicans* and both components at immersion time.

Factorial Plots (pareto chart of the standardization effect, interaction plot and main plots) are publicized in Figs. 8a), b) and c). Figure 8a) demonstrates the normal probability plot of the standardized effects for inhibition efficiency (IE%). Figure 8b) the interaction effect plot for the mean values of (IE%), which gained by DOE, which shows the main effect plot of IE% for Ti alloy in pepsin, *c.albicans* and both components at immersion time. Figure 8c) For immersion time, the IE% decreases till 120 h then increases when reaches 240 h. As for the presence of *candida albicans* only the IE% is high then decreases in the existence of both *candida albicans* and pepsin, the IE% decreased to the minimum. In the presence of pepsin, the IE% increased to the maximum.

## Conclusions

This study demonstrates that the stability of orthodontic Ti-6Al-4 V alloys in pathological oral environments is critically dependent on component interactions. Pepsin alone provides significant, time-enhanced corrosion protection (IE = 87.4%) by forming a protective protein film. However, when co-present with *C. albicans*, pepsin’s proteolytic activity antagonizes the protective microbial biofilm, resulting in a synergistic corrosive effect that drastically lowers protection (IE = 55.6%). The RSM model confirmed that the component interaction is the most significant factor, offering a powerful predictive tool. These findings underscore the clinical importance of managing GERD in orthodontic patients to prevent premature implant failure.

## Data Availability

The datasets used and/or analysed during the current study available from the corresponding author on reasonable request.
